# Health System Integration and Prior Authorization in Medicare Advantage

**DOI:** 10.1111/1475-6773.70137

**Published:** 2026-06-02

**Authors:** Eunhae Shin

**Affiliations:** ^1^ Department of Health Policy and Management University of Georgia Athens Georgia USA

**Keywords:** health system affiliation, Medicare Advantage, prior authorization, utilization management, vertical integration

## Abstract

**Objective:**

To examine whether Medicare Advantage (MA) plans affiliated with health systems adopt less restrictive prior authorization (PA) policies than non‐affiliated plans.

**Study Setting and Design:**

We conducted a descriptive analysis comparing PA policies between system‐affiliated and unaffiliated MA plans from 2016 to 2023. Using Plan Benefit Package data, we constructed enrollment‐weighted measures of PA intensity based on the share of service categories requiring PA. We assessed robustness using alternative measures, including service‐specific PA requirements, service mix‐adjusted overall intensity, and measures without enrollment weights, and also examined results by plan type.

**Data Sources and Analytic Sample:**

Analyses included 6480 MA health maintenance organization and preferred provider organization plans operating during the study period.

**Principal Findings:**

Enrollees in system‐affiliated MA plans faced fewer PA requirements than those in unaffiliated plans. In 2023, the difference was 31 percentage points (pp): among 23 service categories, enrollees in affiliated plans were enrolled in plans that required PA for 51% of categories, compared with 82% in unaffiliated plans. Differences were especially pronounced for behavioral and mental health services and dialysis (52–59 pp).

**Conclusions:**

System affiliation is associated with less restrictive PA policies in MA, highlighting organizational structure as an important dimension of utilization management.

## Abstract (highlights)

**What Is Known on This Topic:**

- Prior authorization (PA) is more prevalent in Medicare Advantage (MA) than in traditional Medicare or Medicaid and has expanded markedly, prompting concerns about administrative burden, delayed care, and access barriers.
- Enrollment in MA has grown rapidly, coinciding with increasing consolidation between MA plans and provider organizations and a growing number of enrollees in MA plans affiliated with health systems.
- Existing research on health system‐affiliated MA plans has documented differences in plan characteristics, quality ratings, and beneficiary satisfaction, but has paid limited attention to utilization management tools such as PA.

**What This Study Found:**

- PA requirements in MA increased through 2020 and then stabilized, with similar trends by affiliation status.
- MA plans affiliated with health systems impose systematically less restrictive PA policies than non‐affiliated plans across multiple measures and samples.
- Differences are especially pronounced for behavioral and mental health services and dialysis, where frequent care may make PA burdensome and potentially disruptive to care continuity in integrated systems.

## 1 Introduction

By 2025, 54% of Medicare beneficiaries are enrolled in Medicare Advantage (MA), a managed care program administered by private insurers that receive capitated payments from Medicare for each enrollee [1]. MA has expanded rapidly, driven by the availability of supplemental benefits such as vision, hearing, and dental coverage; relatively low premiums; annual limits on out‐of‐pocket spending; and extensive marketing [2]. Alongside this growth, consolidation between provider organizations and MA plans has increased, mirroring broader trends in the U.S. health care system. The Medicare Payment Advisory Commission (MedPAC) reports that, in 2023, more than half of MA parent organizations had some degree of health system ownership [3]. MedPAC further notes that such vertical integration may increase plan profitability under current MA payment policy, highlighting the need to monitor these trends and evaluate their implications for beneficiaries and the functioning of the MA program.

To manage spending, MA plans rely on a range of utilization management tools, most notably restrictive provider networks and prior authorization (PA) [4]. PA is the process through which providers obtain insurer approval before delivering certain services and is used more extensively in MA than in traditional Medicare or Medicaid [5, 6]. Nearly all MA plans (99%) impose PA requirements for at least some services, particularly higher‐cost care such as inpatient hospital and skilled nursing facility stays [7]. Although PA is intended to curb low‐value or unnecessary care—potentially enabling additional benefits or lower premiums—it may also create barriers to access, delay clinically appropriate care, and increase unmet need [8, 9, 10, 11]. Importantly, PA use varies substantially across MA insurers and plans [12, 13]. If beneficiaries are differentially exposed to plans with more restrictive PA policies, such variation may unevenly affect access to care and contribute to disparities across populations.

Health system affiliation can fundamentally change how MA plans manage utilization, though the direction of this effect is theoretically ambiguous. Vertical integration partially aligns insurers' and providers' financial incentives and expands organizational capacity for internal coordination—characteristics shown to shape care delivery redesign strategies and the mechanisms through which health systems influence clinical practice [14, 15, 16, 17, 18]. When providers and their affiliated plans share financial risk under capitation, the insurer's need to scrutinize individual service requests ex ante may be reduced. Instead, utilization can be managed through network design, limited availability of higher‐cost therapies within the network, care management protocols, or internal clinical oversight—substitutes for PA that do not require service‐level authorization [19]. In addition, because PA can generate administrative burden for both providers and plans, integrated organizations may have stronger incentives to limit its use, particularly for services that are frequent, ongoing, and operationally complex. At the same time, integration can also streamline rather than eliminate PA by enabling shared information systems and aligned workflows that accelerate approval decisions [20]. These countervailing mechanisms may vary across health systems, which differ in their organizational structure, degree of integration, and the levers used to influence care delivery [21, 22].

Accordingly, integrated plans might either reduce reliance on PA or maintain similar requirements while processing them more efficiently. Consistent with the efficiency channel, a recent study of oncology practices that integrated in‐house specialty pharmacies found shorter time‐to‐fill for prescriptions requiring PA, suggesting that organizational integration can mitigate administrative frictions even when PA requirements formally remain in place [23]. Consistent with the substitution channel, evidence from large MA insurers shows that Kaiser Permanente—a prototypical integrated plan—applies fewer PA and step therapy requirements for costly physician‐administered drugs [19]. Our study extends this literature by providing additional evidence on substitution mechanisms and examining whether health system affiliation is associated with the extent of PA requirements across a broad cross‐section of MA plans and service categories over time. The findings will provide insight into how organizational structure shapes utilization management in the MA market and inform ongoing policy debates on PA reform.

## 2 Methods

### 2.1 Data

This study used the Agency for Healthcare Research and Quality (AHRQ) Compendium of U.S. Health Systems, the most current publicly available longitudinal source of data on health system ownership of MA plans, with data available through 2023 [24]. The Compendium provides information on MA plans owned by health systems in 2016, 2018, and 2020–2023, including unique plan identifiers that allow linkage to publicly available data on MA plan characteristics.

We merged MA plan ownership information from the AHRQ Compendium with Centers for Medicare & Medicaid Services (CMS) Plan Benefit Package (PBP) files for each corresponding year using plan identifiers. The PBP files are submitted annually by MA plans during the bidding process and detail PA requirements across service categories. We further merged CMS Landscape files to incorporate plan characteristics, including monthly consolidated premiums, star ratings, and state and county identifiers. A total of 187 plans appeared only in the Landscape files but not in the PBP files (accounting for approximately 2% of enrollment) and were excluded from the analytic sample. To obtain enrollment counts, we calculated average monthly enrollment per plan‐county‐year using CMS monthly enrollment files from 2016 to 2023. Enrollment counts below 10 are masked and were therefore omitted from the analysis.

We restricted the sample to health maintenance organization (HMO) and preferred provider organization (PPO) plans. We excluded plans limited to specific populations or subject to different payment regulations, including Medicare‐Medicaid plans, Programs of All‐Inclusive Care for the Elderly plans, employer‐sponsored plans, Part B‐only plans, and cost plans [13, 25]. We also excluded UnitedHealthcare, the largest MA insurer, because its PA trends exhibit abrupt shifts from near zero to nearly universal PA between 2019 and 2020. Prior research indicates that these changes primarily reflect reporting artifacts rather than substantive changes in PA practices [13]. The final analytic sample included 6480 unique MA plans offered at any point during the study period.

### 2.2 Measures

The primary exposure was a binary indicator of whether an MA plan was affiliated with a health system. A plan was classified as system affiliated if any non‐federal general acute care hospital within the health system reported in the American Hospital Association Annual Survey Database that it or its system offered an MA plan through ownership or a joint venture, as captured in the AHRQ Compendium [26].

The primary outcome was the average PA rate across 23 service categories at the plan level. Using binary indicators from the PBP files, we calculated the share of service categories requiring PA, where a value of 1 indicated that PA was required and 0 otherwise. We included 17 Medicare‐covered services consistently defined across years in the PBP data and 6 common supplemental benefits spanning dental, vision, and hearing services. We also examined service‐specific PA rates, defined as the percentage of plans requiring PA for each service category.

### 2.3 Empirical Strategy

We conducted descriptive analyses to compare PA policies between system‐affiliated and unaffiliated MA plans. All PA rates were weighted by enrollment to reflect the share of service categories with PA in a typical enrollee's plan (i.e., the overall measure) and the share of enrollees in plans requiring PA for a given service category (i.e., the service‐specific measure).

First, we compared plan characteristics by system affiliation status in 2023 using Landscape and PBP data, including monthly Part C and D consolidated premiums, in‐network maximum out‐of‐pocket amounts, star ratings, and geographic region.

Next, we calculated average annual overall PA rates by system affiliation from 2016 to 2023 to assess temporal trends. As a supplementary analysis, we constructed service mix‐adjusted PA rates, weighting each service category by its share of Medicare fee‐for‐service (FFS) spending using the 2019 CMS Provider Summary by Type of Service files. This approach accounts for the relative fiscal importance of services but yields higher PA rates because PA is more commonly applied to higher‐cost services. It also assumes comparable spending distributions across service categories between FFS Medicare and MA. We, therefore, present unadjusted rates as the primary outcome and use adjusted rates as a robustness check. Details on service categories, procedure codes, spending, and weights are provided in Table S1.

We also examined the share of enrollees subject to PA for each service category in 2023 by affiliation status. Service categories were grouped into eight domains: inpatient and post‐acute care; outpatient diagnostic and radiology services; rehabilitation therapy; behavioral and mental health services; chronic condition‐related services; prescriptions and supplies; ambulance; and supplemental benefits.

Finally, we conducted sensitivity analyses using an alternative measure (without enrollment weights) and alternative samples, restricting to plans with prescription drug coverage and to Special Needs Plans, given differences in benefit design and enrollee characteristics across these plan types.

## 3 Results

Table 1 indicates that, in 2023, approximately 18% of MA enrollees were enrolled in system‐affiliated plans. Enrollees in these plans faced fewer PA requirements than those in non‐affiliated plans. PA rates were 31 percentage points (pp) lower in system‐affiliated plans, implying that, across 23 service categories, enrollees in affiliated plans were enrolled in plans that required PA for 51% of categories, compared with 82% in unaffiliated plans. Service mix‐adjusted PA rates were higher in both groups, with a smaller difference (12 pp), likely reflecting that plans tend to impose PA for higher‐cost services regardless of affiliation status. System‐affiliated plans were also larger and had higher premiums but offered more generous benefits, including lower Part D deductibles and out‐of‐pocket limits, as well as higher average star ratings (4.7 vs. 4.0). In addition, these plans were disproportionately located in the Midwest and more likely to be HMOs; notably, no regional PPOs were observed among system‐affiliated plans, while regional PPOs represented about 1% of plans in the non‐affiliated group. Given their very small share, we do not expect this difference to materially affect the results.

**TABLE 1:** Characteristics of Medicare Advantage plans by health system affiliation, 2023.

	Non‐affiliated MA plans	System‐affiliated MA plans	Total
No. of observations, weighted	14,754,166 (81.8%)	3,285,040 (18.2%)	18,039,206 (100.0%)
PA rate, unadjusted (%)^a^	82 (14)	51 (21)	76 (20)
PA rate, service mix‐adjusted (%)^b^	98 (11)	86 (21)	96 (14)
No. of enrollees per plan	22,471 (31,985)	41,256 (59,546)	25,892 (39,167)
Special Needs Plans (%)	21 (41)	10 (30)	19 (39)
Plans with prescription drug coverage (%)	97 (16)	98 (14)	97 (16)
Monthly consolidated premium (Part C + D) ($)	12 (33)	33 (50)	17 (38)
Annual Part D deductible amount ($)^c^	156 (207)	85 (167)	143 (202)
In network maximum out‐of‐pocket amount ($)	4832 (1881)	4350 (1827)	4735 (1880)
Overall star rating	4.0 (0.67)	4.7 (0.49)	4.1 (0.68)
Plan type			
HMO	2005 (55.7%)	357 (58.5%)	2362 (56.1%)
HMO‐POS	308 (8.6%)	100 (16.4%)	408 (9.7%)
Local PPO	1249 (34.7%)	153 (25.1%)	1402 (33.3%)
Regional PPO	38 (1.1%)	0 (0.0%)	38 (0.9%)
Region			
Midwest	598 (16.6%)	202 (33.1%)	800 (19.0%)
Northeast	584 (16.2%)	112 (18.4%)	696 (16.5%)
South	1574 (43.7%)	150 (24.6%)	1724 (41.0%)
West	844 (23.4%)	146 (23.9%)	990 (23.5%)

*Note:* Sample means are weighted by plan enrollment so that plans with larger enrollment contribute proportionally more. Values are reported as means (standard deviations) for continuous variables and as frequencies (percentages) for categorical variables.

Abbreviations: HMO, health maintenance organization; MA, Medicare Advantage; PA, prior authorization; POS, point of service; PPO, preferred provider organization.

^a^ The prior authorization rate is defined as the percentage of 23 service categories (17 Medicare‐covered services and 6 common supplemental benefits) subject to prior authorization.

^b^ The service mix‐adjusted prior authorization rate is defined as the percentage of 17 Medicare‐covered services subject to prior authorization, weighted by fee‐for‐service Medicare payments for each category.

^c^ Three percent of plans without prescription drug coverage are excluded from this measure.

**FIGURE 1:**
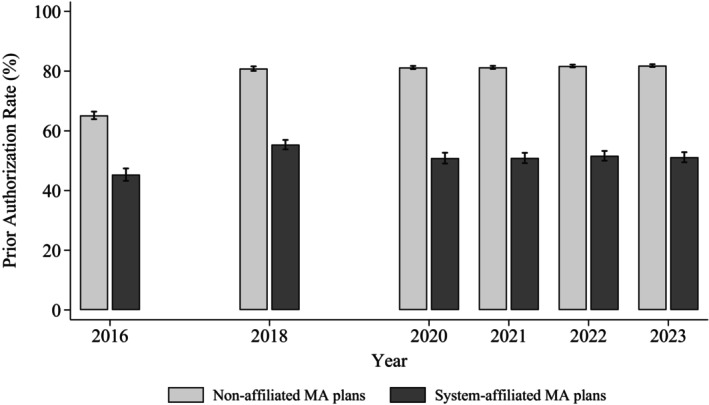
Trends in prior authorization rates by Medicare Advantage plan system affiliation. MA, Medicare Advantage. The prior authorization rate is defined as the percentage of 23 service categories (17 Medicare‐covered services and 6 common supplemental benefits) subject to prior authorization. Rates are averaged across plans within each year by system affiliation status, with plans weighted by enrollment. Error bars denote 95% confidence intervals. Data for 2017 and 2019 are not shown because the Compendium of U.S. Health Systems, the primary data source, is unavailable for those years.

Figure 1 illustrates trends in PA use over time by system affiliation. Both affiliated and non‐affiliated MA plans expanded PA requirements through 2020, after which PA rates stabilized. Throughout the study period, system‐affiliated plans consistently imposed fewer PA requirements, with a persistent gap of 20–31 pp. Consistent with the patterns in Table 1, this gap was smaller when PA rates were weighted by FFS spending: spending‐weighted PA rates were 7–13 pp lower in affiliated plans than in non‐affiliated plans (Figure S1).

**FIGURE 2:**
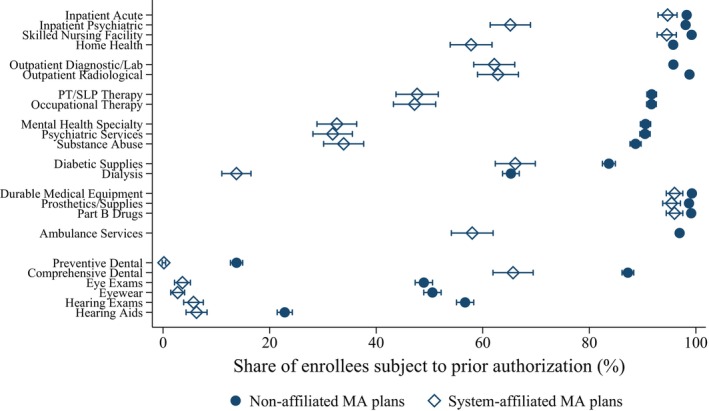
Service‐level prior authorization requirements by Medicare Advantage plan system affiliation, 2023. MA, Medicare Advantage; PT, physical therapy; SLP, speech‐language pathology. The sample includes 4210 MA plans in 2023. Fewer observations are available for preventive dental services (*N* = 4044) and hearing aids (*N* = 3968) because these services are not covered by all plans. Sample means are weighted by plan enrollment so that plans with larger enrollment contribute proportionally more. Error bars denote 95% confidence intervals.

Figure 2 shows PA rates by service category. System‐affiliated plans exhibited lower PA intensity across all categories examined, including supplemental benefits, although the magnitude of the differences varied substantially. For inpatient acute care, skilled nursing facility services, and prescriptions and supplies—including durable medical equipment, prosthetics, supplies, and Part B drugs—PA rates were modestly lower among affiliated plans by 3–5 pp. In contrast, differences were pronounced for behavioral and mental health services, including mental health services, psychiatric services, and outpatient substance use treatment, where PA rates were 55–59 pp lower in affiliated plans. These differences imply that nearly all enrollees in non‐affiliated plans faced PA for behavioral and mental health services, compared with roughly one‐third of enrollees in affiliated plans. Dialysis services showed a similarly large gap, with enrollees in affiliated plans being 52 pp less likely to face PA requirements.

The patterns in Figures 1 and 2 were largely consistent without enrollment weights and within subgroups of plans, including those with prescription drug coverage and Special Needs Plans (Figures S2–S7).

## 4 Discussion

This study shows that MA plans affiliated with health systems impose systematically less restrictive PA policies than non‐affiliated plans. Across multiple measures, system‐affiliated plans required PA for a smaller share of service categories, with larger differences observed when PA rates were not weighted by FFS spending, indicating that divergence in PA practices is concentrated in lower‐cost services. These differences persisted over time and were observed across all service types examined, but were particularly large for behavioral and mental health services and dialysis. These findings suggest that health system affiliation is associated with a distinct approach to utilization management, consistent with theoretical predictions that vertically integrated insurers rely less on PA, as financial incentives are partially internalized and organizational structures provide alternative means of cost control.

The concentration of affiliation‐related differences in behavioral and mental health services and dialysis may reflect the operational complexity and care coordination demands of these services. For conditions that require frequent, ongoing interactions with providers, restrictive PA policies may generate administrative burden, delay care, or disrupt continuity in ways that are especially costly for integrated delivery systems, because these systems internalize both the administrative costs of PA and the downstream consequences of care disruptions. In such settings, affiliated plans may rely more heavily on internal clinical oversight, care pathways, or provider‐level accountability rather than ex ante authorization requirements.

This study builds on prior work using the AHRQ Compendium documenting the characteristics of system‐affiliated MA plans, including their size, geographic distribution, and enrollee composition [24, 27, 28]. These studies generally find that system‐affiliated plans receive higher quality ratings and report greater beneficiary satisfaction than unaffiliated plans. Complementary hospital‐based evidence shows that, among patients hospitalized for myocardial infarction, heart failure, or pneumonia, enrollment in hospital‐owned MA plans and admission to affiliated hospitals are associated with shorter lengths of stay, lower intensive care use, and reduced mortality and readmissions [14]. Our findings point to differences in PA intensity as one potential mechanism underlying these patterns. Although the observed PA differences are concentrated in lower‐cost services, PA requirements may still delay or deter care regardless of a service's fiscal importance; for example, delayed authorization for diagnostic imaging can postpone referrals or treatment initiation. As a result, enrollees without access to system‐affiliated plans may face greater administrative barriers, with potential implications for access, satisfaction, and health outcomes.

This study has several limitations. First, PBP files indicate only whether PA is required for at least one service within broad service categories, not the frequency or service‐specific application of PA. This binary measure performs well for discrete services such as dialysis but is less informative for heterogeneous categories such as Part B drugs, where PA requirements can vary in scope. Second, reporting inconsistencies may introduce measurement error, as illustrated by the UnitedHealthcare case; however, to the extent that such error is not systematically related to system affiliation, it is unlikely to fully explain observed differences between groups. Third, the plan‐level analysis does not directly capture enrollee‐level consequences for access, utilization, or outcomes. Fourth, the study focuses on PA for medical services and does not capture PA under Part D, although medical and pharmacy benefit design is likely jointly determined. Fifth, the descriptive design does not support causal inference regarding the relationship between system affiliation and PA policy design.

Despite these limitations, the findings provide timely insights into policy debates surrounding the evolution of the MA market and PA regulation. Recent discussions emphasize closer insurer‐provider alignment as a mechanism to improve efficiency and advance value‐based care [3, 29]. In this context, health system integration may be associated with reduced reliance on PA, potentially moderating the program‐wide expansion of PA. At the same time, ongoing federal efforts to streamline and regulate PA—such as requirements related to transparency, timeliness, and automation [30]—raise questions about whether integrated and non‐integrated plans differ in their capacity to respond. Understanding how PA reforms differentially affect MA plans and enrollees across organizational structures remains an important area for future research.

## Funding

The author has nothing to report.

## Conflicts of Interest

The author declares no conflicts of interest.

## Supporting information


**Table S1:** Service category definitions and spending weights used in service mix‐adjusted prior authorization rate calculation.
**Figure S1:** Trends in service mix‐adjusted prior authorization rates by Medicare Advantage plan system affiliation.
**Figure S2:** Trends in prior authorization rates by Medicare Advantage plan system affiliation (unweighted by enrollment).
**Figure S3:** Trends in prior authorization rates by Medicare Advantage plan system affiliation (plans with prescription drug coverage only).
**Figure S4:** Trends in prior authorization rates by Medicare Advantage plan system affiliation (Special Needs Plans only).
**Figure S5:** Service‐level prior authorization requirements by Medicare Advantage plan system affiliation (unweighted by enrollment), 2023.
**Figure S6:** Service‐level prior authorization requirements by Medicare Advantage plan system affiliation (plans with prescription drug coverage only), 2023.
**Figure S7:** Service‐level prior authorization requirements by Medicare Advantage plan system affiliation (Special Needs Plans only), 2023.

## Data Availability

The data that support the findings of this study are available in AHRQ Compendium of US Health Systems, at https://www.ahrq.gov/chsp/data‐resources/compendium.html.
