# Supplementary material for: Health System Integration and Prior Authorization in Medicare Advantage
Source: Health Serv Res. 2026 Jun 2;61(3):e70137. doi: 10.1111/1475-6773.70137 (PMC13239139; doi:10.1111/1475-6773.70137)
Supplement: Supplementary file 1 — Table S1: Service category definitions and spending weights used in service mix‐adjusted prior authorization rate calculation. Figure S1: Trends in service mix‐adjusted prior authorization rates by Medicare Advantage plan system affiliation. Figure S2: Trends in prior authorization rates by Medicare Advantage plan system affiliation (unweighted by enrollment). Figure S3: Trends in prior authorization rates by Medicare Advantage plan system affiliation (plans with prescription drug coverage only). Figure S4: Trends in prior authorization rates by Medicare Advantage plan system affiliation (Special Needs Plans only). Figure S5: Service‐level prior authorization requirements by Medicare Advantage plan system affiliation (unweighted by enrollment), 2023. Figure S6: Service‐level prior authorization requirements by Medicare Advantage plan system affiliation (plans with prescription drug coverage only), 2023. Figure S7: Service‐level prior authorization requirements by Medicare Advantage plan system affiliation (Special Needs Plans only), 2023. [file HESR-61-e70137-s001.docx]

# **Supporting Information**

**Table S1. Service category definitions and spending weights used in service mix-adjusted prior authorization rate calculation**

| **Service Category** | **Medicare Payment Source Data** | **Relevant Codes / Definition** | **Total Medicare Payment ($)** | **Assigned Weight** |
| --- | --- | --- | --- | --- |
| Ambulance Services | OP / Physician & Other Practitioners | HCPCS A0225–A0999^a^ | 4,777,590,897 | 0.024 |
| Prosthetics/Medical Supplies | DME | HCPCS L0000–L9999^a^ | 182,492,477 | 0.001 |
| Diabetic Supplies and Services | DME | HCPCS A4206–A4259 and A5500–A5514^a^ | 1,522,423 | < 0.001 |
| Durable Medical Equipment | DME | All remaining HCPCS in DME file after excluding A0225–A0999, L0000–L9999, A4206–A4259, and A5500–A5514^a^ | 65,723,472 | < 0.001 |
| Dialysis Services | OP / Physician & Other Practitioners | HCPCS 90935, 90937, 90940, 90945, 90947, 90951–90970, 90989, 90993, 90997, 90999^b^ | 825,854,253 | 0.004 |
| Medicare Part B Rx Drugs | Physician & Other Practitioners | HCPCS with Part B drug indicator = “Y” (hcpcs_drug_ind = “Y”) | 16,966,092,495 | 0.085 |
| Inpatient Hospital-Acute | IP | All DRG codes in IP except 876 and 880–887 (psychiatric) | 113,925,280,321 | 0.573 |
| Inpatient Hospital Psychiatric | IP | DRG: 876 and 880–887^c^ | 909,333,408 | 0.005 |
| Skilled Nursing Facility | SNF | All services in SNF file | 25,061,084,978 | 0.126 |
| Home Health Services | HHA | All services in HHA file | 17,843,365,188 | 0.090 |
| Occupational Therapy Services | OP / Physician & Other Practitioners | HCPCS 29065–29086; 29105–29131; 29200; 29240; 29260; 29280; 29345; 29365; 29405; 29505–29550; 29799; 90901; 90912–90913; 92526; 92548; 92610; 92626–92627; 95851–95852; 96112–96113; 96125; 97010–97036; 97110–97113; 97124; 97129–97130; 97140; 97150; 97165–97168; 97530; 97533; 97535; 97537; 97542; 97545–97546; 97550–97552; 97597–97598; 97602; 97605–97606; 97750; 97755; 97760–97761; 97763; 97799; G0281; G0283; G0329^d^ | 3,062,574,546 | 0.015 |
| Mental Health Specialty Services | OP / Physician & Other Practitioners | HCPCS 99483; 90785; 90791–90792; 90832–90840; 90845–90849; 90853; 90870; 90880; 90889; 96116, 96121; 96130–96133; 96136–96139; 96146; 96156; 96158–96159; 96161; 96164–96165; 96167–96168; 96170–96171; 96202–96203; 97151–97158; 97550–97552; G0017–G0018; G0136; G0539–G0554; G0560; G2213^e^ | 1,353,767,911 | 0.007 |
|  |  |  |  |  |
| Psychiatric Services | OP / Physician & Other Practitioners | HCPCS 90785; 90791–90792; 90832–90840; 90845–90849; 90853; 90863; 90865; 90870; 90880; 90885; 90887; 90889; 90899; 96105; 96110; 96112–96113; 96116; 96121; 96130–96133; 96136–96139; 96146; G0017–G0018; G0410–G0411; G0451; G0560^f^ | 1,346,223,354 | 0.007 |
| Physical Therapy and Speech-Language Pathology Services | OP / Physician & Other Practitioners | HCPCS 29065–29085; 29105–29131; 29200; 29240; 29260; 29280; 29345; 29365; 29405; 29445; 29505–29580; 29799; 90901; 90912–90913; 92507–92508; 92521–92524; 92548; 92597; 92607–92609; 95851–95852; 95992; 96105; 96112–96113; 96125; 97010–97036; 97110–97116; 97124; 97129–97130; 97140; 97150; 97161–97164; 97530; 97533; 97535; 97537; 97542; 97545–97546; 97550–97552; 97597–97598; 97602; 97605–97606; 97750; 97755; 97760–97761; 97763; 97799; G0281; G0283; G0329^g^ | 3,354,062,849 | 0.017 |
| Outpatient Diagnostic Procedures, Tests, and Lab Services | OP / Physician & Other Practitioners | HCPCS 80047–89999, restricted to outpatient place of service (place_of_srvc = “O”) | 6,472,193,987 | 0.033 |
| Outpatient Substance Abuse | OP / Physician & Other Practitioners | HCPCS G0396–G0397; G2011^e^ | 1,767,123 | < 0.001 |
| Outpatient Diagnostic and Therapeutic Radiological Services | OP / Physician & Other Practitioners | HCPCS 70000–79999, restricted to facility place of service (place_of_srvc = “F”)^h^ | 2,659,735,080 | 0.013 |
| **Total** |  |  | **198,808,664,762** | **1.000** |

NOTES: CMS, Centers for Medicare & Medicaid Services; DME, durable medical equipment; DRG, diagnosis-related group; HCPCS, Healthcare Common Procedure Coding System; HHA, home health agency; IP, inpatient; OP, outpatient; Rx, prescription; SNF, skilled nursing facility.

The source data for total Medicare payments by service category come from CMS’s Provider Summary by Type of Service files, which summarize the use and payments for services provided to Original Medicare (fee-for-service) beneficiaries by inpatient and outpatient hospitals, skilled nursing facilities, home health agencies, physicians, and other health care professionals/suppliers. The total Medicare payment for each category reflects program payments net of beneficiary deductibles and coinsurance. Supplemental benefits (e.g., dental, vision, hearing) are not routinely covered under traditional Medicare and are therefore excluded from the calculation of service mix-adjusted prior authorization rates.

^a^ Gupta, R., Fein, J., Newhouse, J. P., & Schwartz, A. L. (2024). Comparison of prior authorization across insurers: cross sectional evidence from Medicare Advantage. *BMJ*, 384.

^b^ <https://cgsmedicare.com/partb/mr/pdf/esrd.pdf>

^c^ <https://www.cms.gov/icd10m/version372-fullcode-cms/fullcode_cms/P0022.html>

^d^ <https://www.cms.gov/medicare-coverage-database/view/article.aspx?articleid=53064&ver=81&>

^e^ <https://www.cms.gov/files/document/mln1986542-medicare-mental-health-coverage.pdf>

^f^ <https://www.cms.gov/medicare-coverage-database/view/article.aspx?articleId=57130>; <https://www.cms.gov/medicare-coverage-database/view/article.aspx?articleId=57480>; <https://www.cms.gov/medicare-coverage-database/view/article.aspx?articleid=57065&ver=21&>

^g^ <https://www.cms.gov/medicare-coverage-database/view/article.aspx?articleId=53065&ver=70>; <https://www.cms.gov/medicare-coverage-database/view/article.aspx?articleID=54111>

^h^ <https://www.cms.gov/files/document/ncci-policy-manual-2017-chapter-9.pdf>

**Figure S1. Trends in service mix-adjusted prior authorization rates by Medicare Advantage plan system affiliation**

**
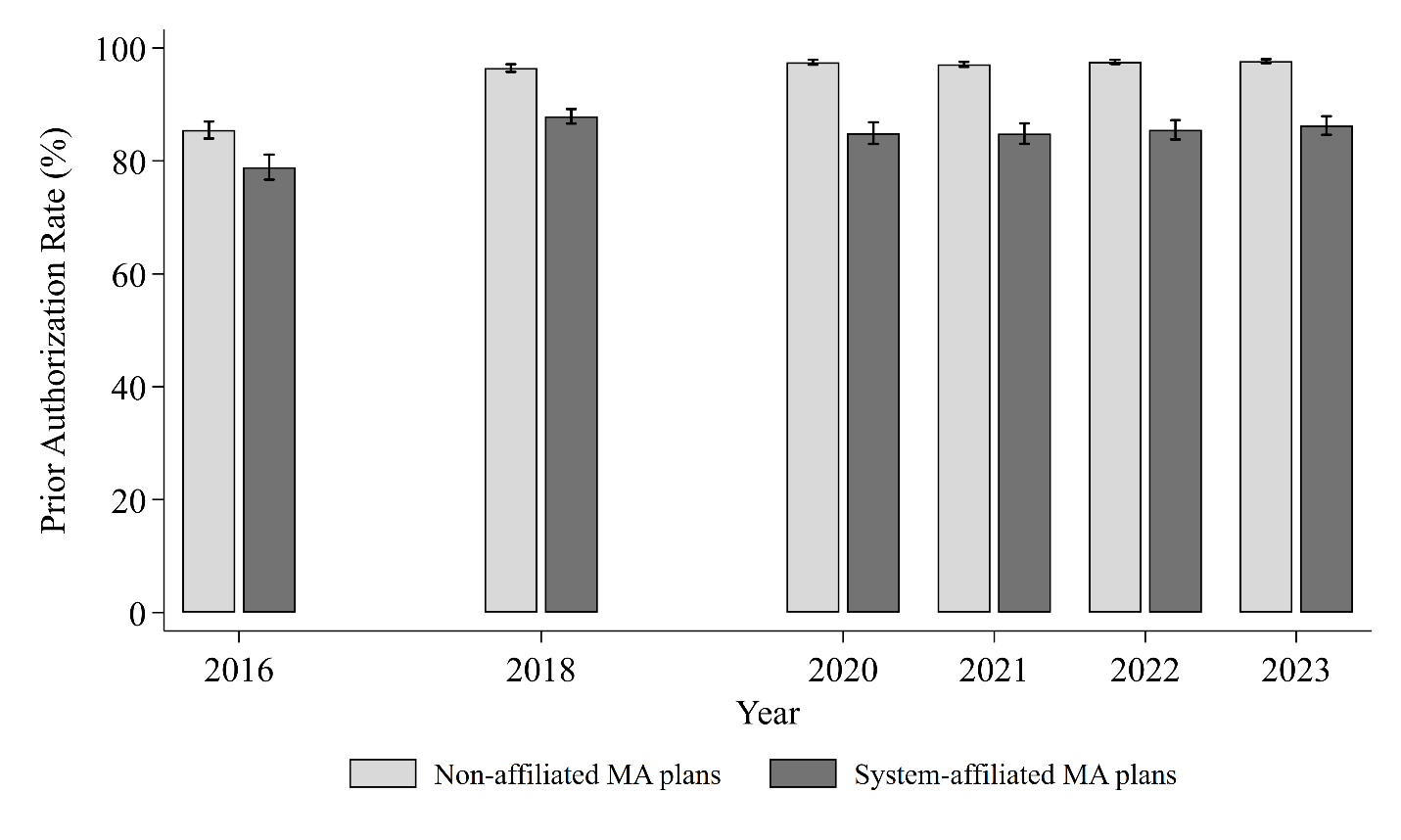
**

NOTES: MA, Medicare Advantage.

The service mix-adjusted prior authorization rate is defined as the percentage of 17 Medicare-covered services subject to prior authorization, weighted by fee-for-service Medicare payments for each category. The 6 supplemental benefits included in the main analysis are excluded because fee-for-service Medicare spending is not defined for these services. Rates are averaged across plans within each year by system affiliation status, with plans weighted by enrollment. Error bars denote 95% confidence intervals. Data for 2017 and 2019 are not shown because the Compendium of U.S. Health Systems, the primary data source, is unavailable for those years.

**Figure S2. Trends in prior authorization rates by Medicare Advantage plan system affiliation (unweighted by enrollment)**

**
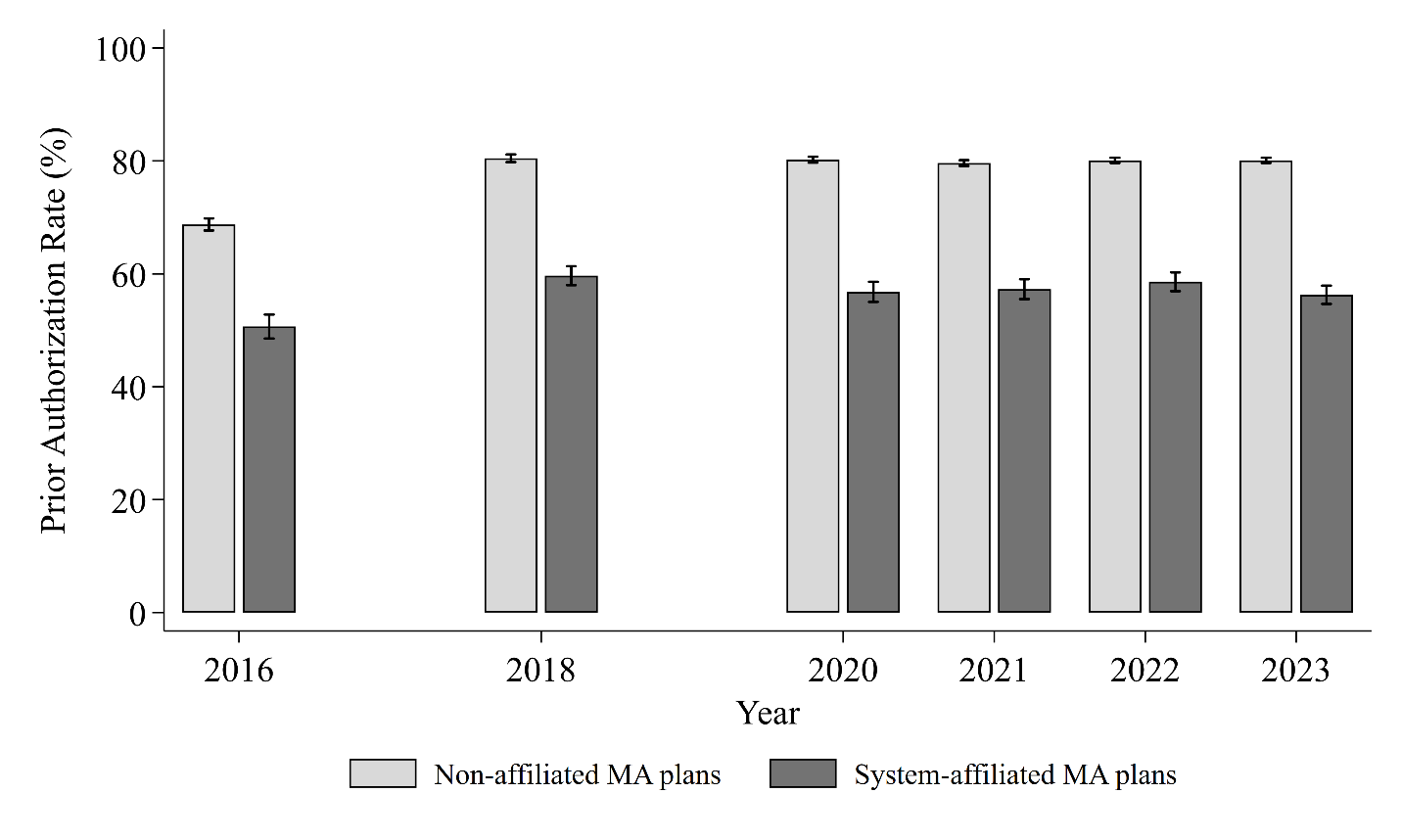
**

NOTES: MA, Medicare Advantage.

The prior authorization rate is defined as the percentage of 23 service categories (17 Medicare-covered services and 6 common supplemental benefits) subject to prior authorization. Rates are averaged across plans within each year by system affiliation status. Error bars denote 95% confidence intervals. Data for 2017 and 2019 are not shown because the Compendium of U.S. Health Systems, the primary data source, is unavailable for those years.

**Figure S3. Trends in prior authorization rates by Medicare Advantage plan system affiliation (plans with prescription drug coverage only)**

**
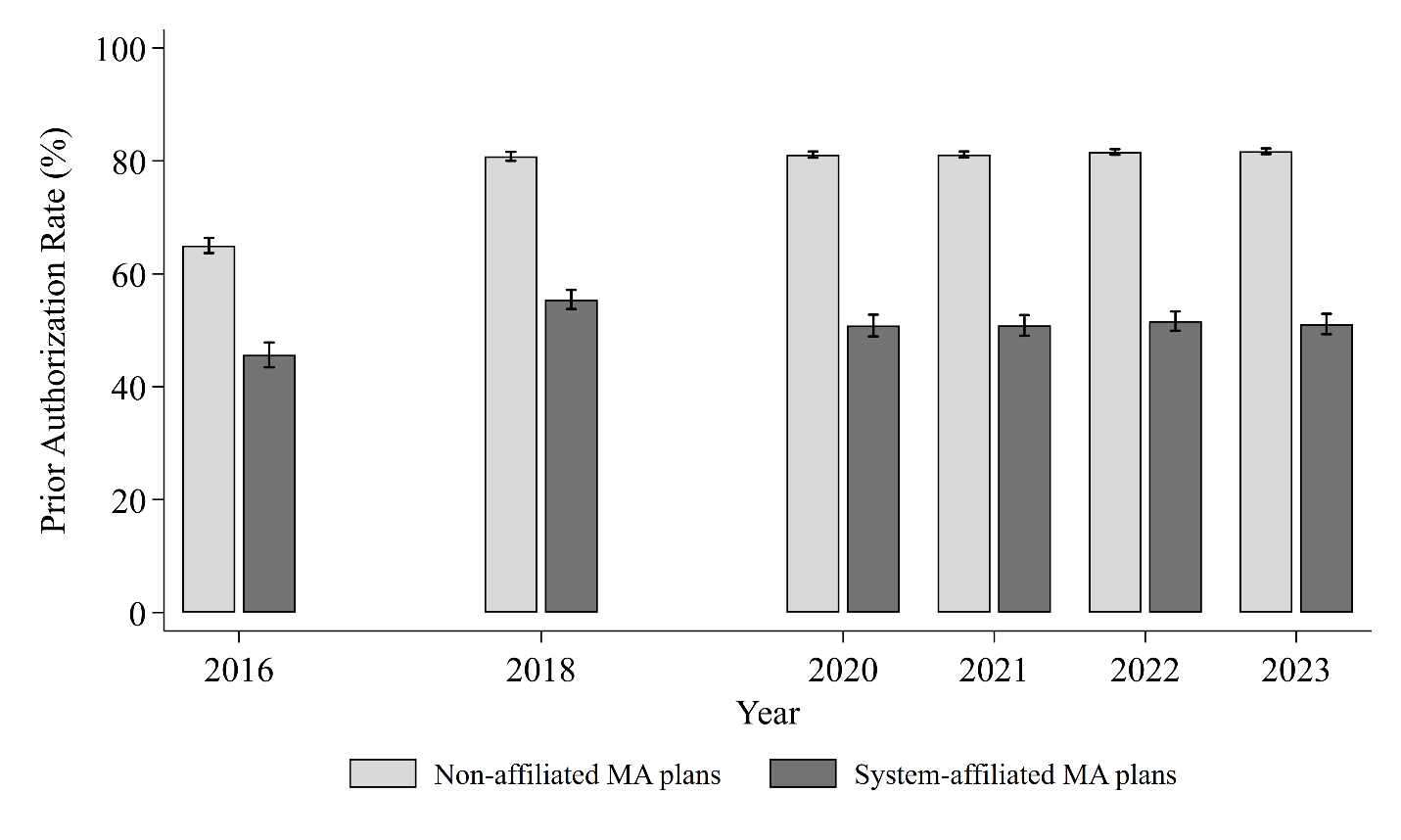
**

NOTES: MA, Medicare Advantage.

The prior authorization rate is defined as the percentage of 23 service categories (17 Medicare-covered services and 6 common supplemental benefits) subject to prior authorization. Rates are averaged across plans within each year by system affiliation status, with plans weighted by enrollment. Error bars denote 95% confidence intervals. Data for 2017 and 2019 are not shown because the Compendium of U.S. Health Systems, the primary data source, is unavailable for those years.

**Figure S4. Trends in prior authorization rates by Medicare Advantage plan system affiliation (Special Needs Plans only)**

**
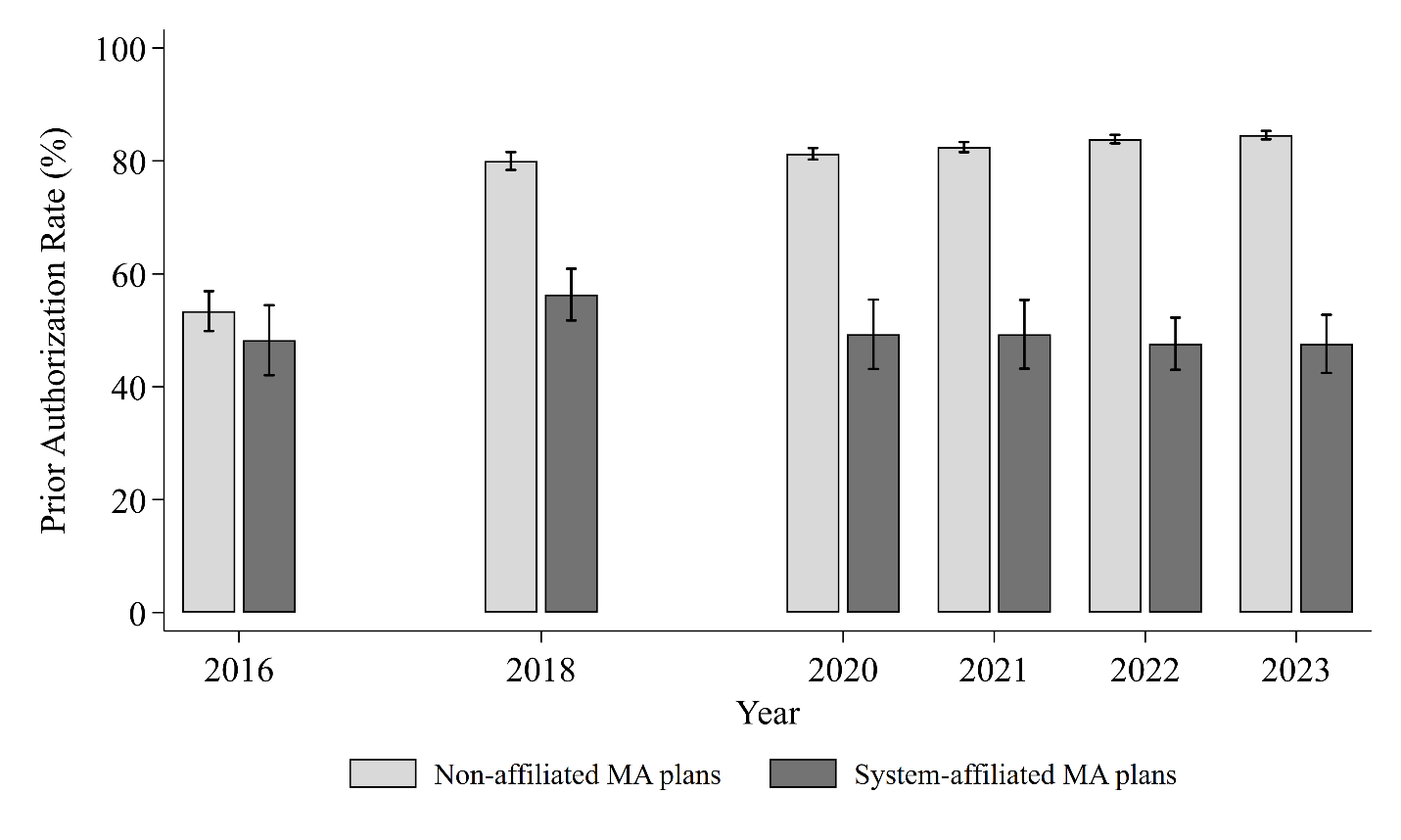
**

NOTES: MA, Medicare Advantage.

The prior authorization rate is defined as the percentage of 23 service categories (17 Medicare-covered services and 6 common supplemental benefits) subject to prior authorization. Rates are averaged across plans within each year by system affiliation status, with plans weighted by enrollment. Error bars denote 95% confidence intervals. Data for 2017 and 2019 are not shown because the Compendium of U.S. Health Systems, the primary data source, is unavailable for those years.

**Figure S5. Service-level prior authorization requirements by Medicare Advantage plan system affiliation (unweighted by enrollment), 2023**
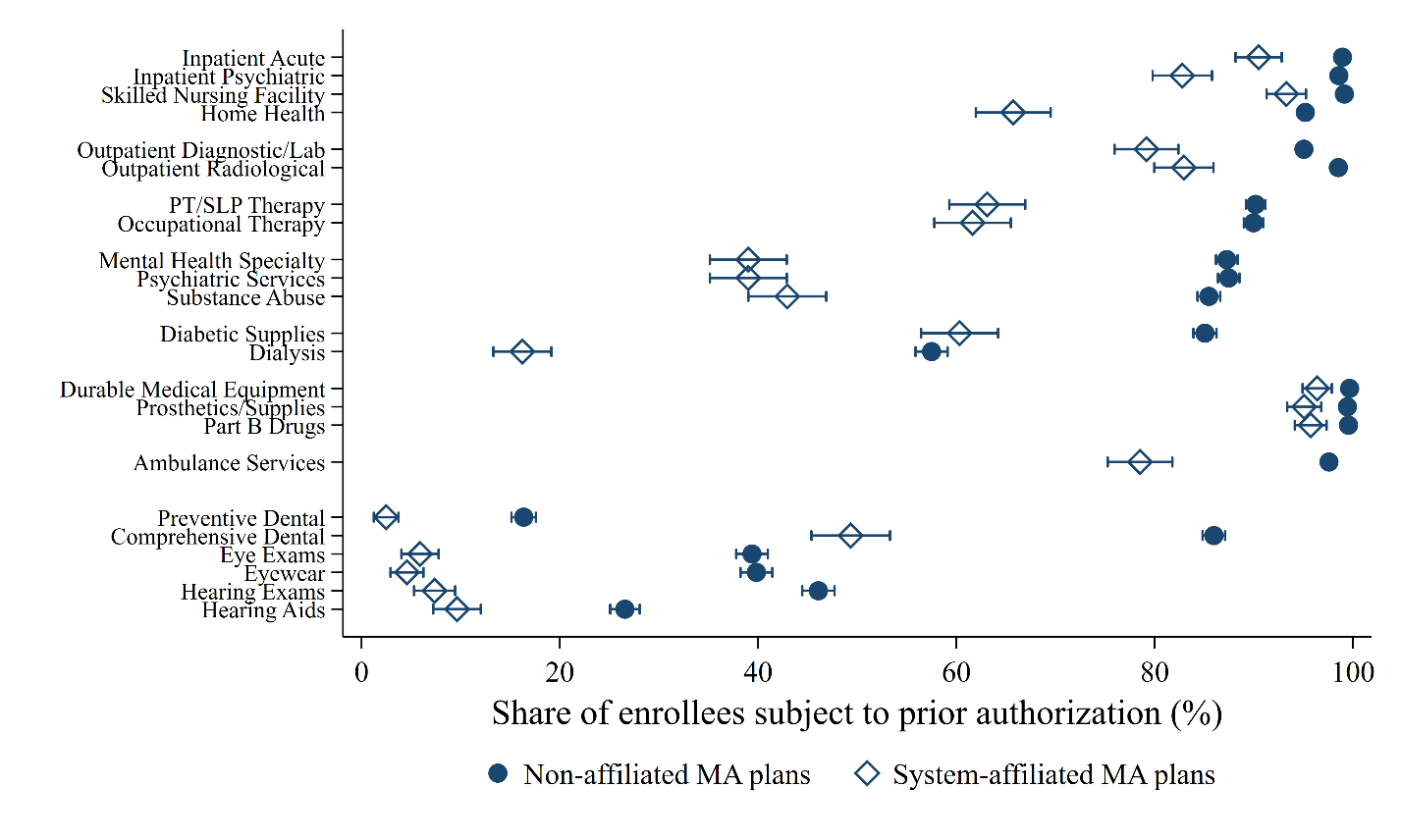
NOTES: MA, Medicare Advantage; PT, physical therapy; SLP, speech-language pathology.

The sample includes 4,210 MA plans in 2023. Fewer observations are available for preventive dental services (N=4,044) and hearing aids (N=3,968) because these services are not covered by all plans. Error bars denote 95% confidence intervals.

**Figure S6. Service-level prior authorization requirements by Medicare Advantage plan system affiliation (plans with prescription drug coverage only), 2023**
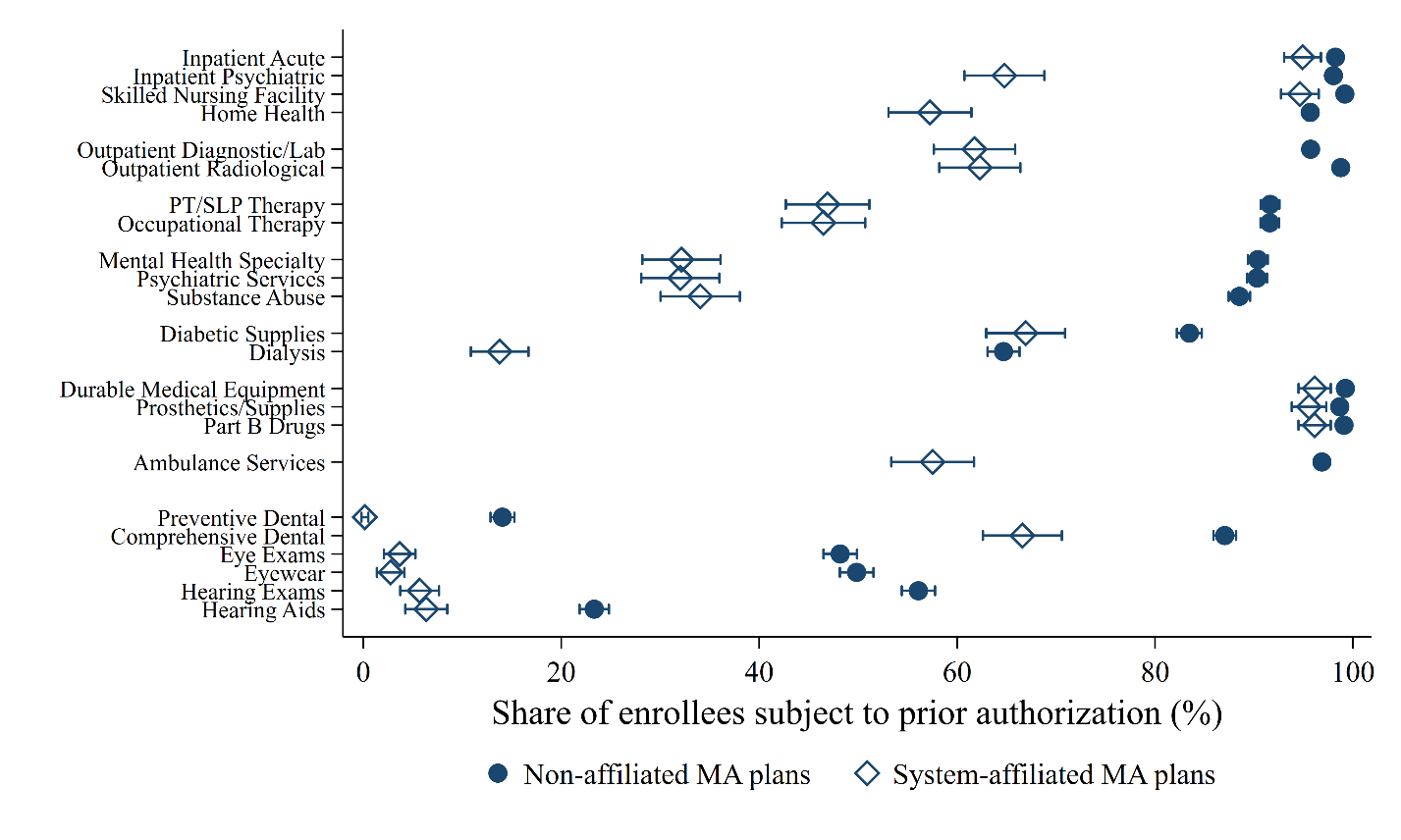
NOTES: MA, Medicare Advantage; PT, physical therapy; SLP, speech-language pathology.

The sample includes 3,881 MA plans in 2023. Fewer observations are available for preventive dental services (N=3,720) and hearing aids (N=3,648) because these services are not covered by all plans. Sample means are weighted by plan enrollment so that plans with larger enrollment contribute proportionally more. Error bars denote 95% confidence intervals.

**Figure S7. Service-level prior authorization requirements by Medicare Advantage plan system affiliation (Special Needs Plans only), 2023**
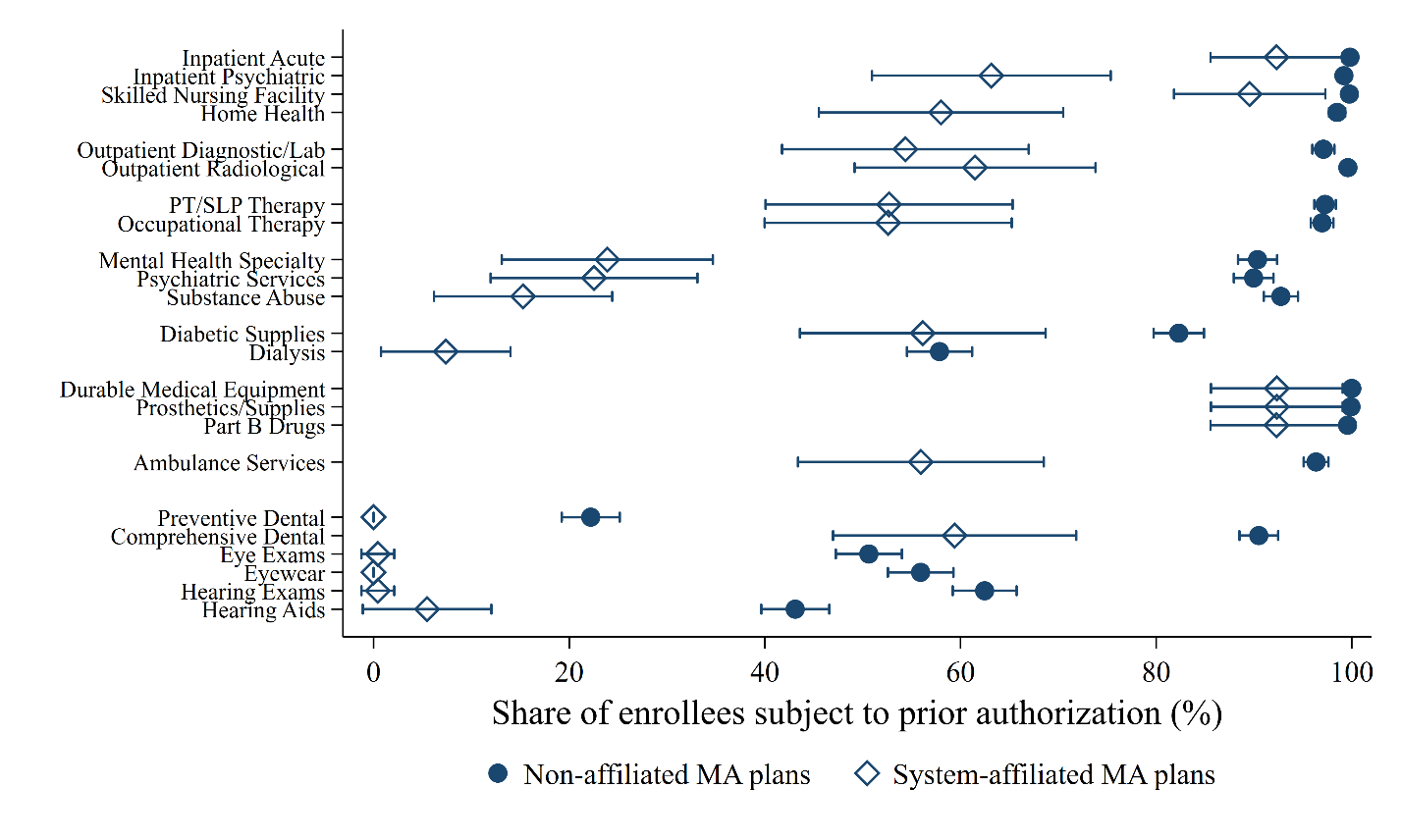
NOTES: MA, Medicare Advantage; PT, physical therapy; SLP, speech-language pathology.

The sample includes 905 MA plans in 2023. Fewer observations are available for preventive dental services (N=816) and hearing aids (N=830) because these services are not covered by all plans. Sample means are weighted by plan enrollment so that plans with larger enrollment contribute proportionally more. Error bars denote 95% confidence intervals.
